# Laser capture microdissection and native mass spectrometry for spatially-resolved analysis of intact protein assemblies in tissue†

**DOI:** 10.1039/d3sc04933g

**Published:** 2024-03-07

**Authors:** James W. Hughes, Emma K. Sisley, Oliver J. Hale, Helen J. Cooper

**Affiliations:** a School of Biosciences, University of Birmingham Edgbaston Birmingham B15 2TT UK h.j.cooper@bham.ac.uk

## Abstract

Previously, we have shown that native ambient mass spectrometry imaging allows the spatial mapping of folded proteins and their complexes in thin tissue sections. Subsequent top-down native ambient mass spectrometry of adjacent tissue section enables protein identification. The challenges associated with protein identification by this approach are (i) the low abundance of proteins in tissue and associated long data acquisition timescales and (ii) irregular spatial distributions which hamper targeted sampling of the relevant tissue location. Here, we demonstrate that these challenges may be overcome through integration of laser capture microdissection in the workflow. We show identification of intact protein assemblies in rat liver tissue and apply the approach to identification of proteins in the granular layer of rat cerebellum.

## Abstract 2

Integration of laser capture microdissection and top-down mass spectrometry aids protein identification in spatially-resolved native ambient mass spectrometry.

## Graphical abstract

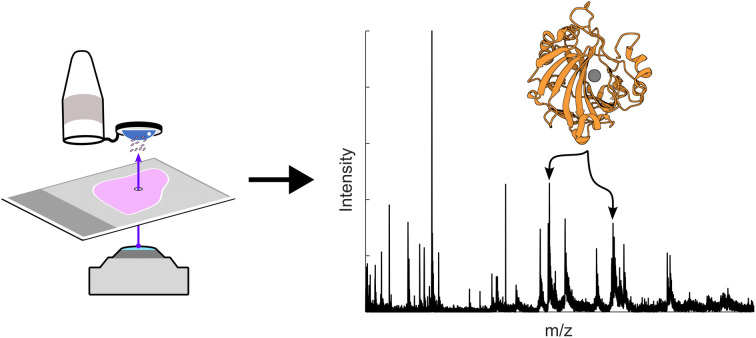

## Introduction

Native ambient mass spectrometry (NAMS) combines native mass spectrometry and ambient mass spectrometry to enable the detection, identification, and spatial mapping of intact proteins and their complexes directly from thin tissue sections. The information available *via* NAMS includes protein sequence (including presence and location of sequence variants and post-translational modifications), assembly or complex stoichiometry, identity of endogenous and exogenous ligands, collision cross sections (when combined with ion mobility spectrometry) and spatial distributions.^1–8^

We have developed NAMS workflows which make use of both liquid extraction surface analysis (LESA)^9^ and nanospray desorption electrospray ionisation (nano-DESI),^10^ which allow *in situ* characterisation of proteins and protein assemblies up to 145 kDa.^11^ Laser ablation electrospray ionisation (LAESI) has also been shown to retain non-covalent interactions but, to date, has only been applied to protein standards.^12^ Native nano-DESI provides higher spatial resolution than LESA (pixel sixes of 200 μm × 200 μm for nano-DESI *vs.* 1 mm × 1 mm for LESA) for native mass spectrometry imaging (MSI). (Other techniques, such as matrix assisted laser desorption ionisation (MALDI), offer higher spatial resolution but are not suitable for native mass spectrometry imaging of proteins^13,14^). Nevertheless, LESA sampling is typically preferred for protein identification as it is compatible with longer data acquisition times (minutes). Protein identification involves dissociation of the intact protein ion in the mass spectrometer to produce sequence fragments whose measured *m*/*z* values are searched against protein databases. Protein ion dissociation is inherently inefficient – the larger the ion, the more degrees of freedom, and the lower the abundance of any individual fragment. This challenge is exacerbated in native mass spectrometry where protein conformation further limits dissociation. While other dissociation techniques, such as ultraviolet photodissociation (UVPD) and surface induced dissociation (SID), are available and could provide complementary information, they are yet to be applied in native ambient mass spectrometry.^15,16^ To obtain high confidence protein assignments, it is therefore necessary to acquire multiple co-added mass spectra, with associated longer acquisition times. Consequently, LESA sampling – either with integrated ionisation or with separate nano-ESI using bespoke tips – is generally preferred for protein identification experiments, as mentioned above; however, the repeatability and accuracy of LESA sampling has been shown to be sub-optimal. Moreover, its poor spatial resolution means it is not suitable for sampling of spatially discrete analytes. Nano-DESI may be employed for protein identification; however, the low absolute signal intensity, coupled with the requirement for movement of the probe to maintain signal, limits its use in this regard. A further limitation is that nano-DESI involves raster sampling across a surface and produces square (or rectangular) pixels. If a protein is distributed across a region that is tangential to the direction of sampling or is otherwise irregularly distributed, targeted sampling of the relevant tissue location may not be possible.

To address these limitations, we sought to investigate laser capture microdissection (LCMD) for spatially-resolved native mass spectrometry of proteins and their complexes in tissue. LCMD is a well-established microscopy technique which makes use of a laser to ablate and catapult material from a surface into a capture vessel.^17^ LCMD has been demonstrated as a powerful technique for bottom-up proteomics where the ability to target specific regions of interest allows single cells and their proteome to be extracted and characterised.^18^ MSI using LCMD has also been achieved in a high throughput and high resolution manner using the nanoPOTS (Nanodroplet Processing in One pot for Trace Samples) platform.^19^ Top-down (TD) proteomics has also been performed using LCMD methods using denaturing solvents. Lubeckyj *et al.* have shown TD LCMD coupled with capillary zone electrophoresis (CZE) for the analysis of zebrafish brain proteoforms wherein regions comprising approximately 250 cells were extracted in a targeted manner.^20^ Liao *et al.* have shown TD LCMD coupled to LC separation yielding ∼500 proteoforms from the cortex and hippocampus of rat brain combined.^21^

Here, we demonstrate integration of LCMD and native mass spectrometry for protein identification directly from tissue. No sample preparation (other than LCMD capture of the material) is employed, aligning with the goal of NAMS. This LCMD workflow is not intended as an imaging workflow but as an aid for spatially-directed identification of proteins. Our results show that intact non-covalent protein complexes, including protein assemblies and protein-metal complexes, can be recovered and characterised from LCMD-excised tissue, with molecular weights ranging from ∼14 kDa to ∼145 kDa. LCMD samples sizes were equivalent or smaller than pixel sizes currently attainable with native nano-DESI sampling. We also demonstrate that LCMD is useful for identification of proteins with irregular spatial distributions by applying the approach to identification of proteins in the granular layer of rat cerebellum.

## Results and discussion

Initial optimisation of the LCMD process was performed to ensure that the laser power was sufficient for a clean dissection of the tissue but did not adversely affect the resulting mass spectra. At higher laser powers, peaks corresponding to protein oxidation and/or abundant singly-charged ions (likely arising from the plastic collection tube) were observed (data not shown).

**Fig. 1:**
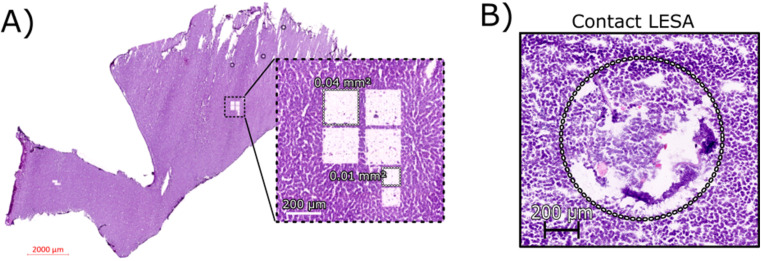
(A) H&E stained section of rat liver tissue post-LCMD analysis. The enlarged region shows 6 separate sampling regions of interest that were obtained. Four larger regions (0.04 mm^2^) and two smaller regions (0.01 mm^2^) are shown cut from a 10 μm thick rat liver section. (B) H&E stained section of rat liver tissue showing the contact LESA sampled region (0.7 mm^2^).

Fig. 1A shows an H&E stained section of rat liver tissue post-LCMD. Six individual regions of interest were dissected with areas of 0.04 mm^2^ or 0.01 mm^2^ (200 μm × 200 μm and 100 μm × 100 μm respectively). The larger LCMD dimensions (200 μm × 200 μm) are equivalent to the pixel sizes typically used in native nano-DESI MSI^7,11^ and are significantly smaller than those obtained by native LESA MSI (1 mm × 1 mm).^3,4,6^ For comparison, Fig. 1B shows a H&E section of rat liver tissue sampled using contact LESA.^22^ The total area sampled in this case is 0.7 mm^2^. (In making these comparisons, it should be noted that both nano-DESI and LESA sampling involve surface sampling of the tissue, whereas LCMD sampling extracts the entire thickness (10 μm) of the tissue).

**Fig. 2:**
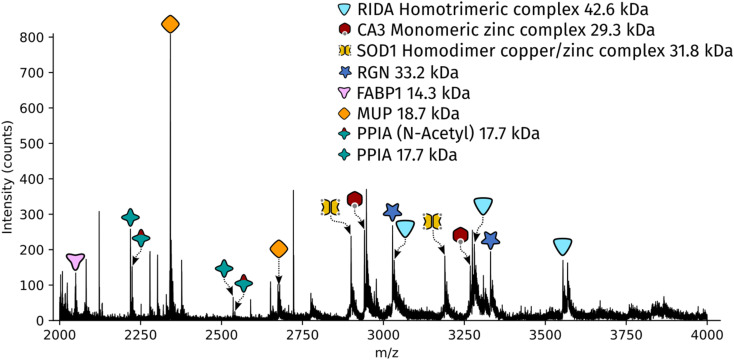
Full scan mass spectrum acquired over 2 minutes (resolution = 120k FWHM at *m*/*z* 200, standard pressure mode) of a 0.01 mm^2^ region of interest captured from a 10 μm thick rat liver section.

Dissected tissue regions were collected in the lid of the collection tube using a small droplet of ammonium acetate. The sample was then transferred into a conductive emitter for nanoelectrospray ionisation (nESI) and native mass spectrometry analysis (full experimental details are given in the ESI†). Representative mass spectra corresponding to the two LCMD sample areas are shown in Fig. S1, ESI.† The mass spectrum obtained from the smaller sample area is very similar to the mass spectrum obtained from the larger region. Fig. 2 shows the full scan mass spectrum acquired from the smaller of the LCMD extraction areas (0.01 mm^2^, *i.e.*, considerably smaller than currently published native nano-DESI MSI and native LESA MSI). The LCMD mass spectrum contains similar peaks to the mass spectrum obtained using nano-DESI (Fig. S2, ESI†). From the LCMD samples, seven proteins, including three protein complexes, were identified by top-down MS/MS. See Table S2, ESI.†

### Identification of protein complexes

Two protein assemblies were identified – the 2-iminobutanoate/2-iminopropanoate deaminase (RidA) homotrimer (42.6 kDa) and the superoxide dismutase 1 (SOD1) homodimer. SOD1 was observed in its holo-form, *i.e.*, each subunit was bound to one zinc ions and one copper ion. A further metal-binding protein was identified – the zinc-bound carbonic anhydrase 3 (CA3).

The protein RidA is an enzyme which catalyses the deamination of imine intermediates in normal metabolism.^23^ RidA has previously been identified in native LESA MSI and native nano-DESI MSI of kidney.^6,7^ Fig. S3, ESI,† illustrates the process by which RidA was identified in the LCMD sample. Firstly, proton transfer charge reduction (PTCR) mass spectrometry was used to determine the intact mass of the protein complex. PTCR involves an ion–ion reaction between the multiply-charged protein cation and a reagent anion which results in proton transfer and a reduction in the charge state of the protein ion.^24^ A series of charge-reduced product ions are generated which allow the intact mass of the protein to be determined from low mass resolution data. Using this approach, the intact mass of the protein was determined to be 42.6 kDa. In a separate mass spectrometry experiment, the stoichiometry of the protein assembly was determined by collisional activation of the 13+ precursor. Product ions corresponding to 6+, 7+ and 8+ subunits with molecular weight 14.2 kDa were observed confirming that the intact assembly exists as a trimer. By further increasing the collision energy, sequence fragments were generated (Table S3, ESI†) which confirm the identity of the protein as RidA.

SOD1 is an antioxidant metalloenzyme that regulates oxidative stress.^25^ It exists as a homodimer in which each subunit binds one Cu^+^ ion and one Zn^2+^ ion. The SOD1 homodimer has previously been detected in native nano-DESI MSI of mouse tissue (manuscript submitted). The protein was identified in the LCMD sample by higher energy collisional dissociation (HCD) (Fig. S4, ESI†). Fragmentation of the precursor (11+ charge state) resulted in dissociation to apo, mono- and dimetal-bound monomer subunits in addition to sequence fragments (see Table S4, ESI†).

CA3 is a zinc-binding enzyme which catalyses the interconversion of CO_2_ and bicarbonate.^26^ A peak corresponding to the 10+ charge state of holo-CA3 was observed in the mass spectra from the LCMD samples. High resolution mass spectrometry confirmed the mass as 29.4 kDa (Fig. S5, ESI†). Precursor ions were subjected to HCD in both the larger (200 μm × 200 μm) and smaller (100 μm × 100 μm) LCMD samples, see Fig. S5, ESI.† A greater number of fragment ions were observed in the larger sample; however, it was possible to confidently identify the protein from both samples (Tables S5 and S6, ESI†).

### Identification of monomeric proteins

In addition to the protein complexes, three monomeric proteins were identified. Fatty acid binding protein 1 (FABP1) is a regulator of hepatic lipid metabolism and is abundant in the liver. It is commonly observed in ambient mass spectrometry of liver sections using both native-like^27^ and denaturing^28,29^ sampling solvents. Its presence was confirmed in the LCMD sample by top-down fragmentation (Fig. S6 and Table S7, ESI†).

Peptidyl-prolyl *cis*–*trans* isomerase A (PPIA) catalyses the *cis*–*trans* isomerization of prolines in proteins and facilitates protein folding. The 17.7 kDa enzyme was detected in the LCMD sample as two proteoforms with a mass difference of 42 Da corresponding to the unmodified and *N*-acetylated protein. Differences in the ratio of unmodified to *N*-acetylated PPIA have been observed in chemo-resistant cells when compared to responsive cancer cells.^30^ Top-down fragmentation of the two proteoforms resulted in the mass spectra shown in Fig. S7, ESI.† This experiment exploited the narrow quadrupole isolation possible with a Q-Exactive HF mass spectrometer to enable the separate isolation of these two proteoforms. The sequence fragments b_28_^2+^ and b_26_^+^ were observed for both proteoforms with mass shifts of 42 Da, consistent with the presence of *N*-acetylation (Tables S8 and S9, ESI†).

The most abundant peak in the mass spectrum shown in Fig. 2 corresponds to the 8+ charge state of major urinary protein (MUP). MUPs are involved in regulation of social behaviour through the binding and release of pheromones and are predominantly produced in the liver.^31^ MUP has been detected previously in native LESA and native nano-DESI experiments.^6,7^ The identity of the 18.7 kDa protein was confirmed by top-down fragmentation (Fig. S8 and Table S10, ESI†).

Regucalcin (RGN) monomer was also identified by top-down fragmentation from a liver extraction (Fig. S9 and Table S11, ESI†). RGN is involved in the regulation of intracellular Ca^2+^ concentration. RGN has also been previously detected from kidney using native nano-DESI.^7,32^

Here, we have been able to show that proteins can be detected and identified using a native LCMD workflow. The majority of these proteins have been identified previously in our native nano-DESI analysis of various tissues and we have noted no evidence of truncations of proteins or disruption of non-covalent interactions with this LCMD approach. The spatial specificity of this LCMD workflow combined with the enhanced electrospray stability achieved with pulled tips should prove to be a complementary technique for native protein identification. The proteins presented here are abundant proteins in the liver but due to the nature of LCMD analysis (*i.e.*, decoupling of sampling from ionisation), further separation steps, such as size exclusion, could also be incorporated to improve depth of coverage.

### Analysis of protein complexes >100 kDa

We have previously demonstrated that, through the inclusion of detergents (specifically octyl tetraethylene glycol ether, C8E4) in the nanoelectrospray solvent, careful tuning of source optics, and optimisation of chamber pressures, larger (>100 kDa) protein complexes can be detected from thin tissue sections by native nano-DESI mass spectrometry^11^ We therefore sought to translate that approach to the LCMD samples. Addition of detergent to the extraction solution prior to LCMD altered the surface tension of the extraction droplet, changing its shape, which complicated the LCMD procedure. Instead of forming a droplet in the centre of the sample collection cap, the addition of the detergent caused the droplet to spread to the edges of the cap and consequently was less efficient at capturing the tissue sample. To overcome this, detergent was added to the sample after LCMD extraction but before nanoelectrospray. The resulting mass spectrum is shown in Fig. S10A, ESI.† Automated deconvolution of this mass spectrum by use of the UniDec software^33^ revealed the presence of two dominant proteins with molecular weights 145.5 kDa and 160.5 kDa (Fig. S10B, ESI†). The mass of the 145 kDa protein was confirmed by PTCR (Fig. S11, ESI†) and is assigned on the basis of intact mass as the homotetramer l-lactate dehydrogenase A (LDHA) which has previously been observed by native nano-DESI mass spectrometry.^11^

### Analysis of the granular layer in the cerebellum of the brain

The approach outlined here has the potential to be particularly useful for identification of proteins with irregular spatial distributions that do not lend themselves to the grid-like sampling and square pixels of nano-DESI or LESA. To investigate that potential, LCMD coupled with native mass spectrometry was applied to the granular layer of the cerebellum in rat brain. Native nano-DESI MSI is capable of achieving the spatial resolution required to detect proteins in the granular layer (width ∼ 200 μm); however, it is irregularly shaped making top-down identification of proteins unique to this area challenging.

**Fig. 3:**
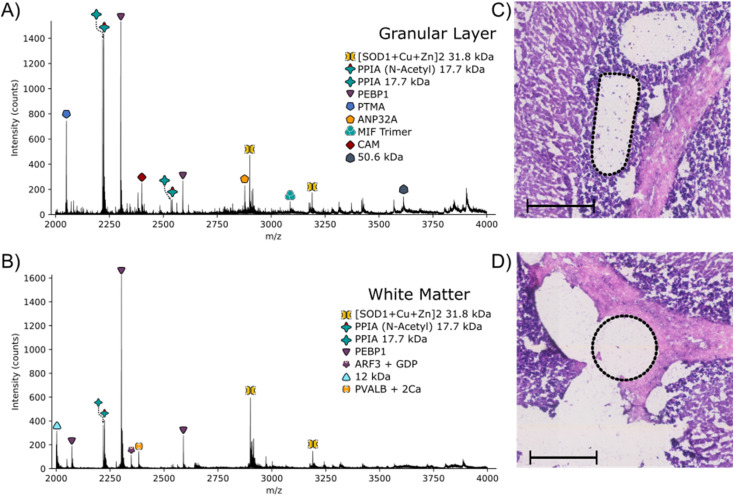
Full scan mass spectrum obtained following LCMD in the granular layer (A) and white matter (B) of the cerebellum of a 10 μm thick rat brain section. Laser captured regions from the granular layer (C) and white matter (D) are shown in the H&E images by the dotted black regions. Scale bars = 200 μm.

Regions from the white matter and granular layer in the rat cerebellum were captured by LCMD. Fig. S12, ESI† shows an optical image of the tissue prior to LCMD with the region in the granular layer to be captured defined, together with the H&E stained section after LCMD. The white matter, grey matter and granular layer are labelled. The regions described here can be distinguished by use of bright field microscopy. It is not possible to stain tissue (*e.g.*, with H&E) prior to LCMD as the reagents would cause disruption of native complexes. In cases where regions cannot be distinguished by bright field microscopy, autofluorescence microscopy could be used or alternatively serial sections could be stained. Fig. 3 shows representative mass spectra from the granular layer and white matter along with the H&E stained tissue showing the dissected regions. (Note, some of the tissue has been disrupted in the staining process). The area extracted in the granular layer is irregularly shaped and in the white matter is circular. In both cases, the area of tissue extracted was 0.03 mm^2^, comparable to that of a nano-DESI pixel used in native MSI.

The majority of proteins observed in the mass spectra have been identified previously^3,11^ and have been assigned based on intact mass here. Two peaks are observed in the granular layer mass spectrum in greatly increased abundance compared to the mass spectrum from the white matter. Top-down MS/MS revealed that these peaks corresponded to prothymsin-α (PTMA) and acidic (leucine-rich) nuclear phosphoprotein 32 family member A (ANP32A) (Fig. S13, S14, Tables S12 and S13, ESI†). PTMA is a 12.6 kDa protein and is involved in cell proliferation, division and survival.^34^ Previous immunohistochemistry experiments have revealed that there is a significant increase in abundance of PTMA in the granular layers of the cerebellum and hippocampus,^34^ in agreement with our findings. ANP32A, a 28.5 kDa protein involved in the formation, extension and branching of neurites along with transcriptional regulation, has also been shown to have higher expression in the granular layer of the cerebellum.^35^

## Conclusions

The results demonstrate the integration of LCMD for native top-down MS/MS of proteins from liver and brain tissue. It was possible to detect and identify proteins and their assemblies from extraction dimensions of 100 μm × 100 μm (×10 μm depth). In addition, the approach enabled identification of two proteins from the irregularly-shaped granular layer of rat cerebellum, which have not previously been identified by native ambient mass spectrometry. Our findings suggest that LCMD provides a complementary approach to nano-DESI and LESA (both with integrated and decoupled ionisation) for identification of proteins detected in native ambient mass spectrometry imaging. Future work could focus on improving throughput by transferring the dissected regions into a well plate and use of a native MS infusion cart^36^ to automate the MS analysis.

## Data availability

Supplementary data supporting this research is openly available from https://doi.org/10.25500/edata.bham.00001043.

## Author contributions

J. W. H – conceptualization, investigation, writing – original draft; E. K. S. – investigation, writing – original draft, review & editing; O. J. H. – investigation, writing – review & editing; H. J. C. – funding acquisition, writing – review & editing.

## Conflicts of interest

There are no conflicts of interest.

## Supplementary Material

SC-015-D3SC04933G-s001
