# Supplementary material for: Laser capture microdissection and native mass spectrometry for spatially-resolved analysis of intact protein assemblies in tissue
Source: Chem Sci. 2024 Mar 7;15(15):5723–9. doi: 10.1039/d3sc04933g (PMC11023061; doi:10.1039/d3sc04933g)
Supplement: SC-015-D3SC04933G-s001 [file SC-015-D3SC04933G-s001.pdf]

Electronic Supplementary Material (ESI) for Chemical Science.
This journal is © The Royal Society of Chemistry 2024

Supporting Information for:

Laser capture microdissection and native mass spectrometry for spatially-resolved analysis of intact protein assemblies in tissue

James W. Hughes, Emma K. Sisley, Oliver J. Hale, and Helen J. Cooper

School of Biosciences, University of Birmingham, Edgbaston, Birmingham B15 2TT, UK.

Table of contents

Experimental details

Table S1: Haematoxylin and eosin staining procedure

Figure S1: Representative mass spectra from LCMD extractions

Table S2: Proteins identified following top-down fragmentation from LCMD extractions of rat liver

Figure S2: Comparison of a nano-DESI mass spectrum and an LCMD-extracted mass spectrum

Figure S3: Identification of the RidA trimer

Table S3: Sequence ions observed for the RIDA monomer

Figure S4: Identification of SOD1 dimer

Table S4: Sequence ions observed for the SOD1 monomer

Figure S5: Identification of Zn-bound CA3

Table S5: Sequence ions observed for the CA3 Zn^{2+} complex from a 0.04 mm^2 ROI

Table S6: Sequence ions observed for the CA3 Zn^{2+} complex from a 0.01 mm^2 ROI

Figure S6: Identification of FABP1

Table S7: Sequence ions observed for FABP1

Figure S7: Identification of proteoforms of PPIA

Table S8: Sequence ions observed for unmodified PPIA

Table S9: Sequence ions observed for N-acetylated PPIA

Figure S8: Identification of MUP

Table S10: Sequence ions observed for MUP

Figure S9: Identification of regucalcin (RGN)

Table S11: Sequence ions observed for RGN

Figure S10: Mass spectrum of proteins >100 kDa from rat liver

Figure S11: PTCR mass spectrum of LDHA monomer

Figure S12: Optical images of rat brain cerebellum before and after LCMD sampling

Figure S13: Identification of ANP32A

Table S12: Sequence ions observed for ANP32A

Figure S14: Identification of PTMA

Table S13: Sequence ions observed for PTMA

Experimental Details

Materials

MS grade water was purchased from Fisher Scientific (Loughborough, UK). HPLC grade ammonium acetate was purchased from J.T. Baker (Deventer, Netherlands). Calibration solutions were purchased from Thermo Fisher Scientific (San Jose, CA). The solvent system used for capture of LCMD material and for subsequent electrospray was 200 mM aqueous ammonium acetate. For analysis of >100 kDa proteins, detergent (C8E4) was added to the extraction solvent after LCMD extraction at 0.5x its critical micelle concentration. Nitrogen (>99.995 %) and helium (>99.996%) gases used within the mass spectrometer were obtained from BOC (Guilford, UK). Harris hematoxylin, acid alcohol, industrial denatured alcohol, Scott's tap water substitute, xylene, and eosin (1% aqueous) were purchased from pfm Medical (Cheshire, UK). DPX was purchased from Cellpath (Powys, UK).

Animal Tissues

Vehicle-dosed (0.5% HPMC and 0.1% Tween 80 in water) liver and brain tissue from adult male Hans Wistar rats was the kind gift of Prof Richard Goodwin. The animal was euthanised 2 h post dose and dissection was performed by trained AstraZeneca staff (project licence PP77366793, procedure number 3). All procedures were conducted in accordance with the United Kingdom Animal (Scientific Procedures) Act 1986, approved by institutional ethical review committee (Babraham Institute Animal Welfare and Ethical Review Board) and conducted under Project Licence authority. The liver and brain were snap frozen in isopentane over dry ice. All tissues were stored at -80 ^{o}C. Sectioning was performed at -22 ^{o}C on a CM1520 cryotome (Leica Microsystems, Wetzlar, Germany) and the 10 µm tissue sections were thaw mounted on to clean glass microscope slides. Slide mounted tissue was stored in a -80 ^{o}C freezer until use. Tissue washing and other sample preparation techniques were not employed to prevent delocalisation of the analytes. Tissue was allowed to thaw and dry in a vacuum desiccator for at least 15 minutes before LCMD to prevent the development of condensation on the tissue surface.

Laser capture microdissection (LCMD)

LCMD was performed with a Zeiss PALM MicroBeam (Carl Zeiss Microscopy, Jena, Germany). The slide-mounted tissue was loaded into the LCMD sample plate. A 20x magnification was used for all LCMD experiments. A region of interest was defined either using a fixed geometry shape or drawn freehand around desired features. All regions of interest (ROI) were first cut out to provide an outline and a cut edge between the bulk tissue and the ROI. Once a region was cut out, the collection tube with ~10 µL of 200 mM ammonium acetate as the collection droplet was repositioned over the tissue and a new ROI was superimposed over the previous using the AUTOLPC cutting function which provides the catapulting laser pulse. The laser energies used were optimised each time such that the minimum energy required to lift the tissue from the slide was used. Collected samples were spun in a benchtop centrifuge for ~ 1-2 s to transfer the extraction solution from the cap of the microcentrifuge tube to the base of the tube to prevent potential sample loss when opening the cap.

Electrospray ionisation

Nano-ESI was performed using borosilicate glass emitters (I.D. 0.68 mm O.D. 1.2 mm) which were prepared in-house using a P-1000 pipette puller (Sutter instruments) before coating with gold using a sputter coater (Agar Scientific Ltd.). Nano-ESI voltages typically ranged between 0.8-1.2 kV and were achieved without backing pressure.

Mass spectrometry

Mass spectrometry was performed on an Orbitrap Eclipse mass spectrometer equipped with the HMR^n option. The mass spectrometer was calibrated using FlexMix and the transfer capillary temperature was set to 275 ^oC. The ion routing multipole (IRM) was operated in standard (8 mTorr) for the majority of the analyses. High (20 mTorr) pressure mode for the analysis of the higher molecular weight proteins from the liver (>100 kDa) with the higher pressures in the IRM assisting in the trapping of large ions and the preservation of gas-phase non-covalent interactions. Source fragmentation was employed to assist in the desolvation of ions. When operating the instrument in the high-pressure mode the source CID compensation scaling factor was optimised concurrently with the source fragmentation to tune the m/z window transmitted. The orbitrap analyser was operated with a resolving power of 120,000 (standard pressure mode) or 7500 (high pressure mode) at m/z 200. For the brain tissue analysis, a resolving power of 240,000 at m/z 200 was used.

Proton transfer charge reduction (PTCR) MS^n was performed using perfluoroperhydrophenanthrene (CAS 306-91-2) reagent anion within the high-pressure region of the linear ion trap. Reagent ion automatic gain control target was $2x10^5$, the reaction time was varied between 1-5 ms. Injection time was 500-1500 ms. Isolation of ions was performed using the linear ion trap. The isolation windows for PTCR MS^n experiments were between m/z 10-20.

Higher collision energy dissociation (HCD) was performed on the Eclipse MS for all proteins (except PPIA). Isolation was performed in the linear ion trap using a 15 m/z window. Normalised collision energies (NCE) used are stated with the fragmentation spectrum of each protein.

A Q-Exactive HF mass spectrometer was used for the MS^n experiments of acetylated and non-acetylated PPIA. The orbitrap analyser was operated with a resolving power of 120,000 . Isolation was performed in the quadrupole using a 5 m/z isolation window to isolate each of the PPIA proteoforms

Protein database searching

ProsightPC 4.1 was used for database searching of fragment ions for top-down identification. The reference Rattus Norvegicus (UP000002494) proteome was downloaded from Uniprot.

H&E staining

Post-laser capture tissue sections were stained using haematoxylin and eosin staining as described below (Table S1). Thaw mounted tissue sections were sequentially submerged in solvent baths containing the listed solvent for the proscribed length of time. Where repeat submersions were required a fresh solvent bath was used. Tissue was mounted in DPX and a cover slip was applied.20x brightfield images were acquired using a Zeiss SlideScanner (Carl Zeiss Microscopy, Jena, Germany).

Table S1: Haematoxylin and eosin staining procedure.

Solvent Bath	Time (min)	Repeat
Water	2	1
Haemotoxylin Harris	4	0
Water	2	1
Acid Alcoholc	0.5	0
Water	2	1
Scott's tap Water Substitute	0.5	0
Water	2	1
Eosin	1	0
Water	2	1
Industrial denatured alcohol	2	3
Xylene	2	2
Mount in DPX	N/A	N/A

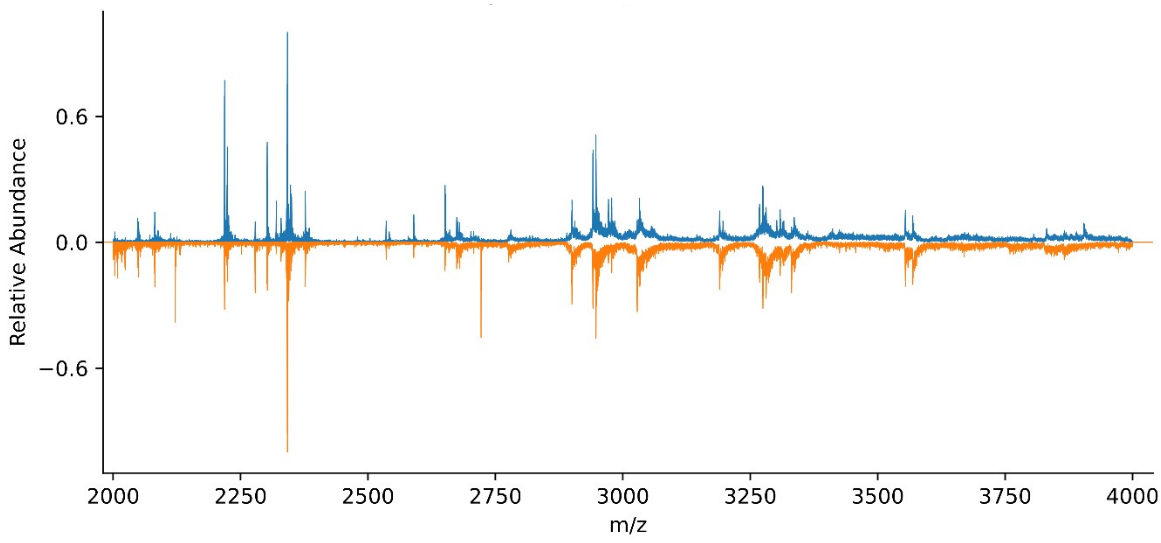

Figure S1: Representative mass spectra from a 200 µm x 200 µm x 10 µm LCMD extraction (blue trace) and a 100 µm x 100 µm x 10 µm LCMD extraction (orange trace). Each mass spectrum comprises ~60 scans.

Table S2: Proteins identified following top-down fragmentation from LCMD extractions of rat liver

Protein	UniProt	PTMs	Experimental mass	Theoretical Mass	Mass Difference (PPM)	Extraction Voxel (μm^3)
FABP1	P02692	N-Acetyl	14305.29	14305.31	1.40	200 x 200 x 10
MUP	P02761	-signal peptide, Disulphide	18716.33	18716.32	0.53	100 x 100 x 10
PPIA	P10111	-Methionine	17731.89	17731.77	6.77	200 x 200 x 10
PPIA	P10111	-Methionine, N-Acetyl	17773.82	17773.79	1.69	200 x 200 x 10
SOD1	P07632	-Methionine, N-Acetyl, Cu(II), Zn(II), Disulphide	31871.12	31871.38	8.16	200 x 200 x 10
RIDA	P52759	-Methionine, N-Acetyl	14205.47	14205.53	4.22	200 x 200 x 10
CA3	P14141	-Methionine, N-Acetyl, Zn	29387.53	29387.07	15.65	200 x 200 x 10<br>100 x 100 x 10
RGN	Q03336	N-Acetyl	33279.55	33279.35	6.01	100 x 100 x 10

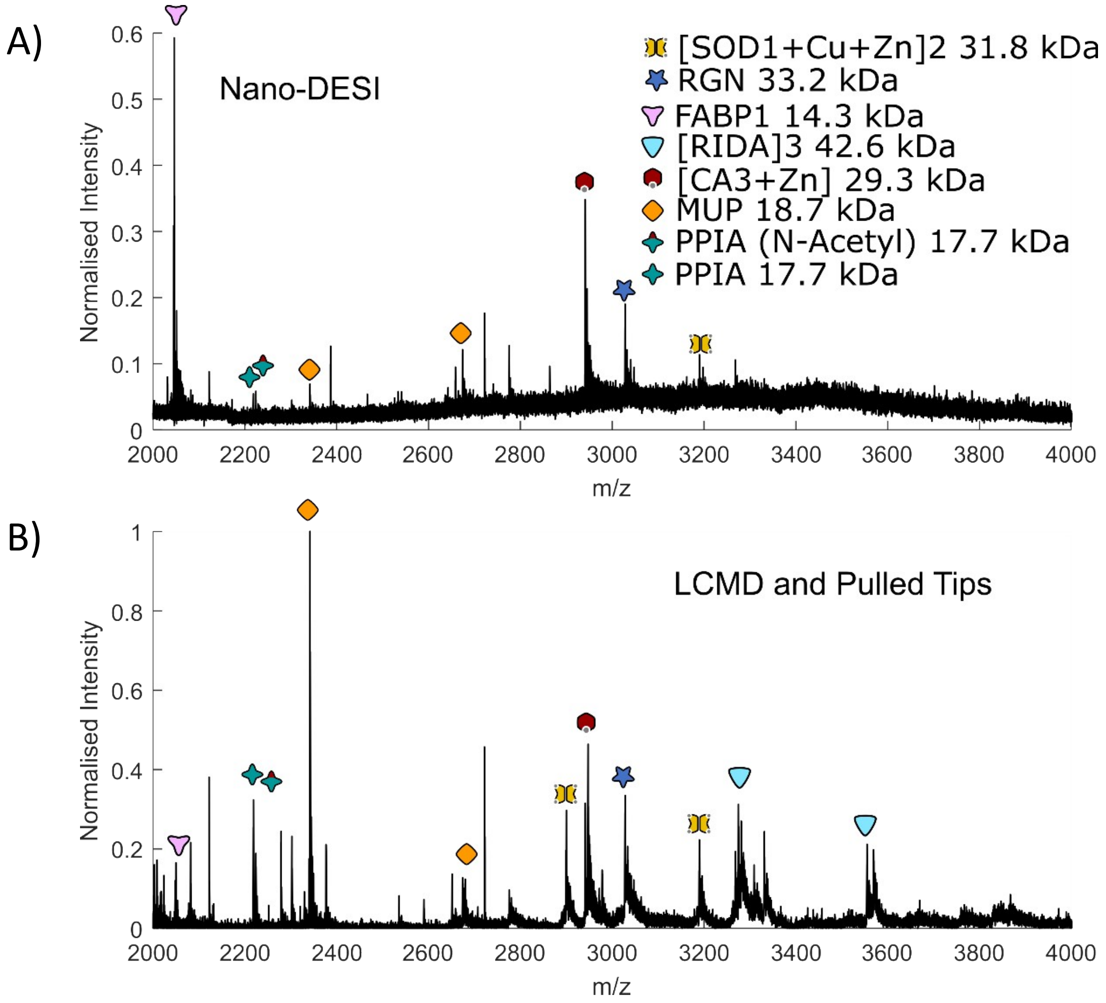

Figure S2: Comparison of mass spectra obtained using A) nano-DESI (line scan, acquired across 6 mins) and B) LCMD capture and nESI using pulled tips (data acquired for 6mins from a 100 x 100 um section) from 10 um thick liver tissue sections.

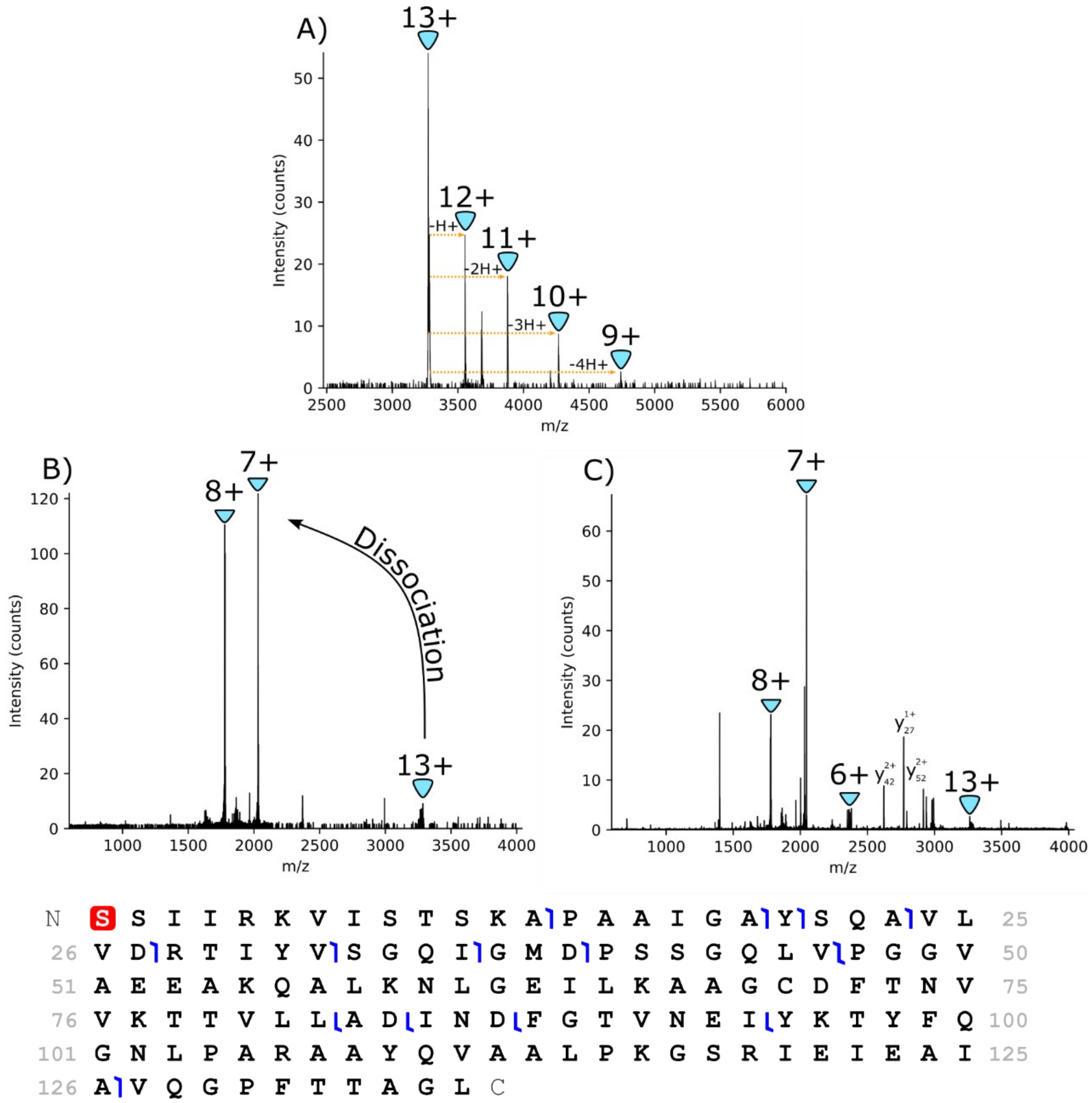

Figure S3: Identification of the RIDA trimeric complex. A) PTCR of the 13+ RIDA complex (isolated in the ion trap using a m/z 15 isolation window and PTCR reaction time of 15 ms) produced a series of charge-reduced peaks which allowed for the determination of the intact complex mass using deconvolution. B) HCD of the 13+ RIDA complex showing dissociation of the complex into monomer subunits. C) HCD (NCE 35%) of the RIDA complex shows fragmentation of the monomer subunits to produce sequence fragments, data acquired for 6 mins. D) Sequence coverage of the RIDA. The red square indicates acetylation.

Table S3: Sequence ions observed for the RIDA monomer

Identity	Experimental Mass (Da)	Theoretical Mass (Da)	Error (Da)	Error (PPM)
b13	1412.8318	1412.8351	-0.003	-2.3
b13	1412.8348	1412.8351	0.000	-0.2
b19	1893.1004	1893.1047	-0.004	-2.3
b20	2056.1510	2056.1680	-0.017	-8.3
b23	2342.2695	2342.2958	-0.026	-11.2
b27	2768.5500	2768.5436	0.007	2.3
b32	3400.8742	3400.9082	-0.034	-10.0
b36	3786.0924	3786.1043	-0.012	-3.1
b36	3786.1099	3786.1043	0.006	1.5
b39	4089.1527	4089.1932	-0.041	-9.9
b39	4089.1790	4089.1932	-0.014	-3.5
b46	4757.5134	4757.5425	-0.029	-6.1
b46	4757.5742	4757.5425	0.032	6.7
b126	13215.8793	13216.0103	-0.131	-9.9
y42	4480.3475	4480.3906	-0.043	-9.6
y49	5240.7092	5240.7662	-0.057	-10.9
y52	5582.8496	5582.9201	-0.071	-12.6
y54	5769.0042	5768.9841	0.020	3.5
y90	9447.9715	9447.9859	-0.015	-1.5

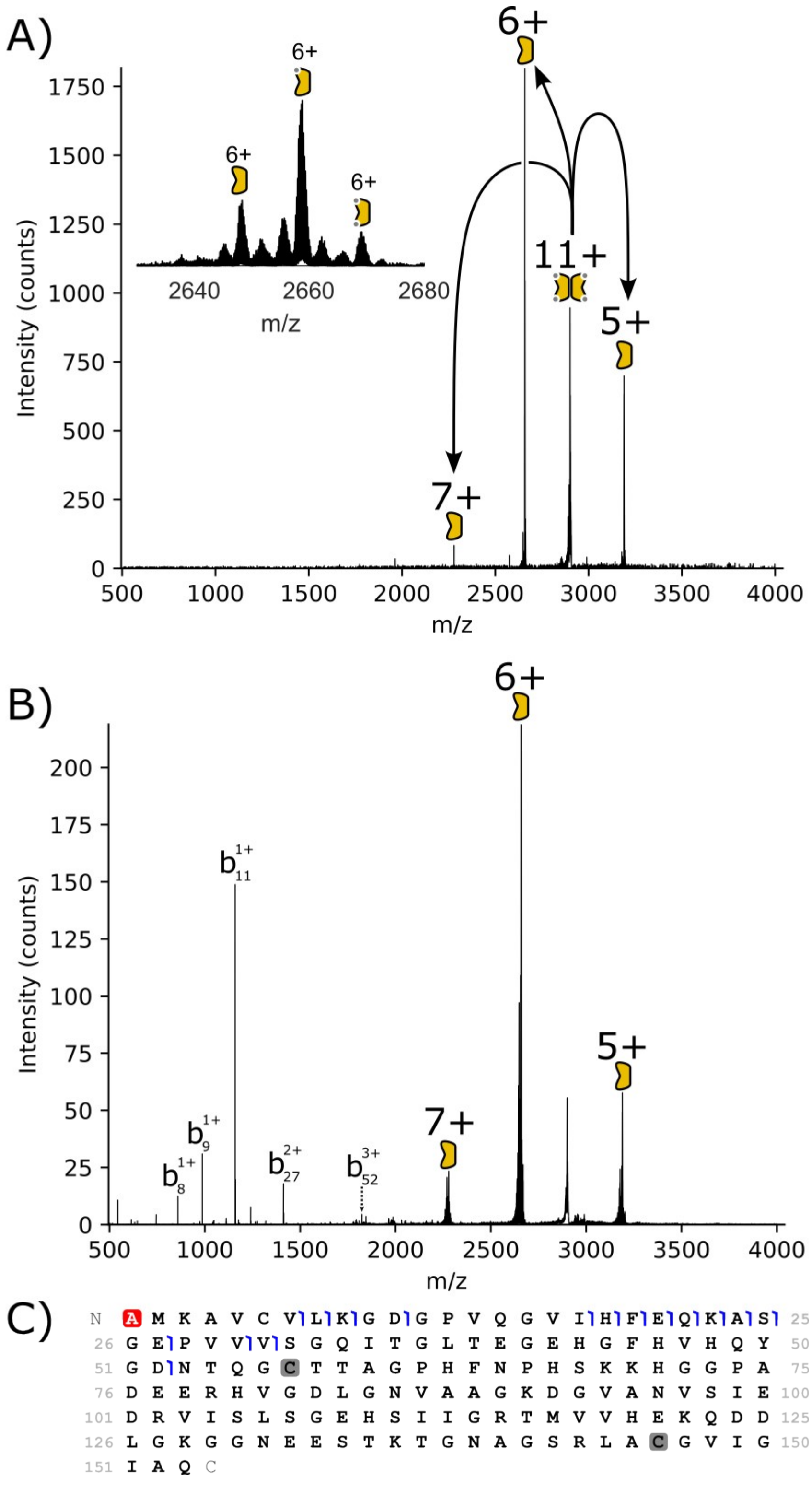

Figure S4: Identification of SOD1 dimer from a 200 µm x 200 µm x10 µm region LCMD extract. A) HCD (NCE 35%) of SOD1, inset shows the apo, mono- and dimetal-bound 6+ monomer subunits B) HCD (NCE 45%) of the SOD1, data acquired for 30 mins. C) Sequence coverage of the SOD1. The red square indicates N-acetylation and grey squares indicate disulphide bond.

Table S4: Sequence ions observed for the SOD1 monomer

Identity	Experimental Mass (Da)	Theoretical Mass (Da)	Error (Da)	Error (PPM)
b7	745.3695	745.3735	0.0040	5.4
b8	858.4529	858.4576	0.0047	5.5
b19	973.5160	973.5212	0.0052	5.3
b9	986.5473	986.5526	0.0053	5.4
b30	1039.8724	1039.8790	0.0066	6.3
b20	1047.0495	1047.0554	0.0059	5.6
b31	1072.8969	1072.9018	0.0049	4.6
b21	1111.5751	1111.5767	0.0016	1.4
b11	1158.5957	1158.6010	0.0053	4.6
b22	1175.5997	1175.6059	0.0062	5.3
b23	1239.6464	1239.6534	0.0070	5.6
b24	1275.1648	1275.1720	0.0072	5.6
b25	1318.6799	1318.6880	0.0081	6.1
b27	1411.7122	1411.7200	0.0078	5.5
b18	1808.9664	1808.9761	0.0097	5.4
b52	1822.8958	1822.9048	0.0090	4.9

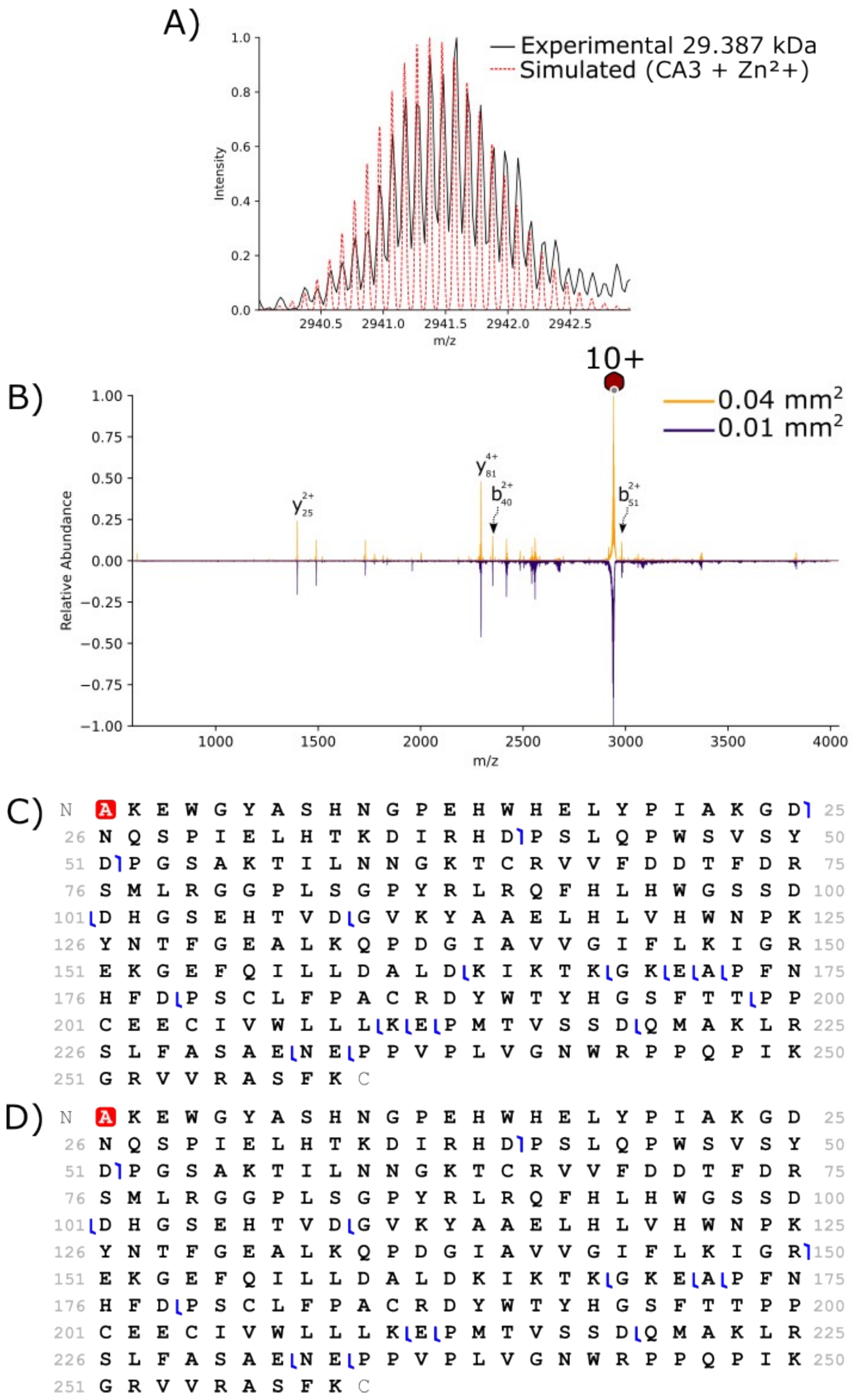

Figure S5: identification of Zn-bound CA3. A) Experimental (solid black line) and theoretical (red hashed line) isotope distributions for the 10+ charge state of the CA3 zinc (II) metal complex. B) Comparison between the HCD (NCE 40%) fragmentation spectrum of CA3 acquired for ROIs 200 µm x 200 µm x10 µm (top, orange line) and 100 µm x 100 µm x10 µm (bottom, purple line). Each mass spectrum was acquired for 6 minutes. C) Sequence coverage of the CA3 protein following HCD MS from a 200 µm x 200 µm x10 µm LCMD sample. D) Sequence coverage of the CA3 protein following HCD MS from a 100 µm x 100 µm x10 µm LCMD sample.

Table S5: Sequence ions observed for holo-CA3 complex from a 200 µm x 200 µm x10 µm LCMD sample.

Identity	Experimental Mass (Da)	Theoretical Mass (Da)	Error (Da)	Error (PPM)
b25	2915.3236	2915.3415	-0.0179	-6.2
b40	4699.1874	4699.2380	-0.0506	-10.8
b51	5958.7537	5958.8203	-0.0666	-11.2
y25	2794.6175	2794.6234	-0.0059	-2.1
y27	3037.6942	3037.7089	-0.0147	-4.8
y40	4470.4217	4470.4586	-0.0369	-8.2
y40	4470.4311	4470.4586	-0.0275	-6.2
y47	5187.7282	5187.7589	-0.0307	-5.9
y48	5316.7668	5316.8015	-0.0347	-6.5
y49	5444.9012	5444.8965	0.0047	0.9
y61	6840.5625	6840.5895	-0.0270	-4.0
y81	9173.4821	9173.5929	-0.1108	-12.1
y81	9173.5880	9173.5929	-0.0049	-0.5
y81	9173.6001	9173.5929	0.0072	0.8
y87	9930.8078	9930.9113	-0.1034	-10.4
y88	10001.9527	10001.9484	0.0044	0.4
y89	10130.9109	10130.9910	-0.0801	-7.9
y91	10316.0318	10316.1074	-0.0756	-7.3
y96	10914.4755	10914.5240	-0.0485	-4.4
y150	16916.6635	16916.7489	-0.0854	-5.1
y159	17894.0316	17894.1328	-0.1012	-5.7

Table S6: Sequence ions for the holo CA3 from 100 µm x 100 µm x 10 µm LCMD sample.

Identity	Experimental Mass (Da)	Theoretical Mass (Da)	Error (Da)	Error (PPM)
b150	2420.6786	2420.6366	-0.042	17.4
b40	2350.6179	2350.6263	0.0084	3.6
b51	2980.4057	2980.4175	0.0118	4.0
y150	2417.6754	2417.6857	0.0103	4.3
y159	2557.2986	2557.3120	0.0134	5.2
y25	1398.3130	1398.3190	0.006	4.3
y27	1519.8557	1519.8618	0.0061	4.0
y40	1491.1533	1491.1602	0.0069	4.6
y47	1730.2523	1730.2603	0.008	4.6
y48	1773.2657	1773.2745	0.0088	5.0
y81	2294.3823	2294.4055	0.0232	10.1
y87	2483.7042	2483.7351	0.0309	12.4
y88	2501.5577	2501.4944	-0.0633	25.3
y91	2580.0150	2580.0342	0.0192	7.4

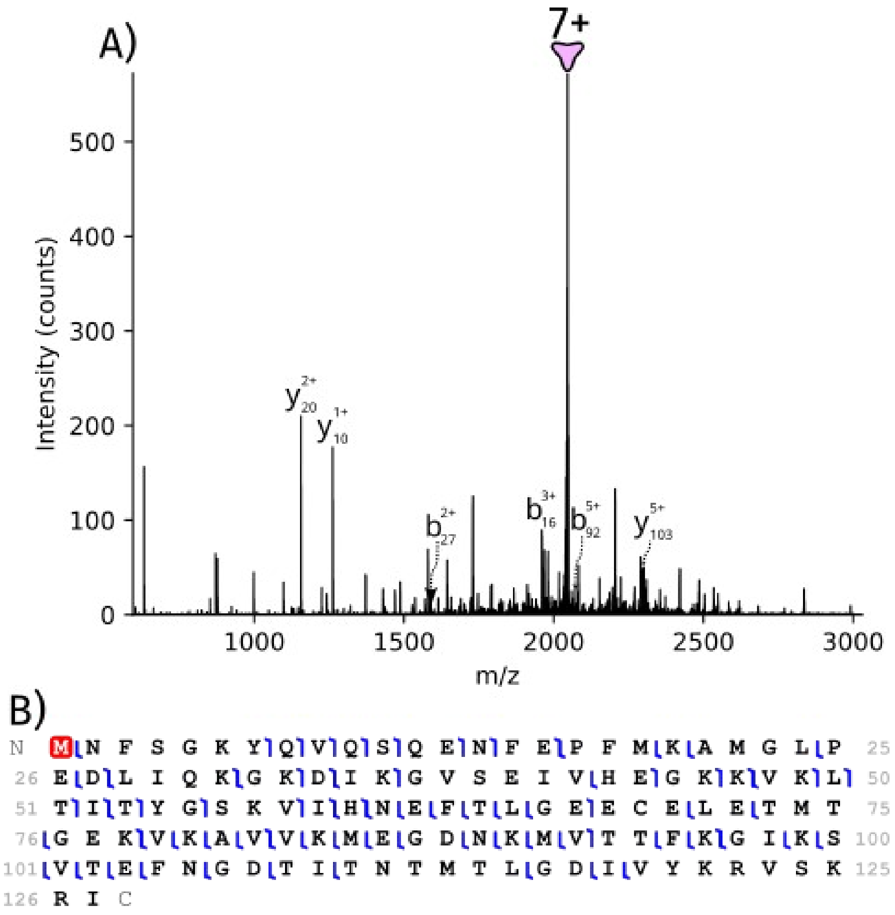

Figure S6: Identification of FABP1. A) HCD (40% NCE) of the 7+ charge state of FABP1, data acquired for 6 mins. B) Sequence coverage observed for FABP1. The red square indicates N-acetylation.

Table S7: Sequence ions observed for FABP1.

Identity	Experimental Mass (Da)	Theoretical Mass (Da)	Error (Da)	Error(PPM)
b7	869.3747	869.3742	0.0006	0.6
b8	997.4346	997.4328	0.0019	1.9
b9	1096.5019	1096.5012	0.0008	0.7
b10	1224.5625	1224.5597	0.0027	2.2
b11	1311.5912	1311.5918	-6E-04	-0.4
b13	1568.6899	1568.6929	-0.003	-1.9
b14	1682.7362	1682.7359	0.0003	0.2
b16	1958.8469	1958.8469	0	0.0
b27	3175.3759	3175.4089	-0.033	-10.4
b31	3657.7359	3657.7306	0.0054	1.5
b33	3842.8277	3842.847	-0.019	-5.0
b34	3957.8534	3957.8739	-0.021	-5.2

b36	4199.048	4199.0529	-0.005	-1.2
b44	5049.4732	5049.4714	0.0018	0.4
b46	5234.524	5234.5878	-0.064	-12.2
b47	5362.6692	5362.6828	-0.014	-2.5
b49	5589.7889	5589.8462	-0.057	-10.2
b50	5702.9216	5702.9302	-0.009	-1.5
b51	5803.9788	5803.9779	0.0009	0.2
b52	5916.9864	5917.062	-0.076	-12.8
b53	6018.1147	6018.1096	0.005	0.8
b55	6238.151	6238.1944	-0.044	-7.0
b58	6552.3501	6552.3898	-0.04	-6.1
b59	6665.4378	6665.4739	-0.036	-5.4
b60	6802.4757	6802.5328	-0.057	-8.4
b67	7592.8111	7592.8825	-0.072	-9.4
b78	8843.4198	8843.3984	0.0214	2.4
b82	9240.6704	9240.6673	0.0031	0.3
b92	10372.1841	10372.2089	-0.025	-2.4
b96	10849.4381	10849.4677	-0.03	-2.7
y9	1147.7164	1147.7189	-0.003	-2.2
y10	1260.8005	1260.803	-0.003	-2.0
y12	1432.8495	1432.8514	-0.002	-1.3
y18	2094.161	2094.1619	-9E-04	-0.4
y20	2308.2928	2308.2937	-9E-04	-0.4
y22	2480.3317	2480.3421	-0.01	-4.2
y24	2741.4568	2741.4534	0.0034	1.2
y25	2870.5035	2870.496	0.0075	2.6
y26	2971.5583	2971.5437	0.0147	4.9
y27	3070.6195	3070.6121	0.0074	2.4
y28	3157.6464	3157.6441	0.0023	0.7
y29	3285.7058	3285.7391	-0.033	-10.1
y31	3455.8126	3455.8446	-0.032	-9.3
y32	3583.9481	3583.9396	0.0085	2.4
y33	3730.9843	3731.008	-0.024	-6.3
y36	4032.1742	4032.1717	0.0024	0.6
y37	4163.224	4163.2122	0.0118	2.8
y38	4291.2774	4291.3072	-0.03	-6.9
y39	4405.3271	4405.3501	-0.023	-5.2
y39	4405.3467	4405.3501	-0.003	-0.8
y41	4577.3922	4577.3985	-0.006	-1.4
y42	4706.4478	4706.4411	0.0067	1.4
y43	4837.4268	4837.4816	-0.055	-11.3
y43	4837.4531	4837.4816	-0.029	-5.9
y44	4965.5689	4965.5766	-0.008	-1.5
y45	5064.659	5064.645	0.014	2.8

y46	5163.6727	5163.7134	-0.041	-7.9
y47	5234.7592	5234.7505	0.0087	1.7
y48	5362.8521	5362.8454	0.0067	1.2
y49	5461.8645	5461.9139	-0.049	-9.0
y52	5776.0448	5776.0729	-0.028	-4.9
y55	6109.14	6109.2087	-0.069	-11.2
y57	6351.3035	6351.3354	-0.032	-5.0
y62	6898.4834	6898.4938	-0.01	-1.5
y63	7011.5012	7011.5778	-0.077	-10.9
y64	7112.6309	7112.6255	0.0054	0.8
y65	7259.5861	7259.6939	-0.108	-14.9
y66	7388.7136	7388.7365	-0.023	-3.1
y67	7502.6724	7502.7795	-0.107	-14.3
y68	7639.8072	7639.8384	-0.031	-4.1
y75	8388.2004	8388.2503	-0.05	-6.0
y80	8942.6706	8942.6295	0.0411	4.6
y85	9521.8805	9521.9423	-0.062	-6.5
y93	10347.3991	10347.4383	-0.039	-3.8
y96	10647.5547	10647.5817	-0.027	-2.5
y100	11129.8538	11129.9033	-0.05	-4.5
y101	11244.8056	11244.9303	-0.125	-11.1
y103	11471.0214	11471.0256	-0.004	-0.4
y107	11843.1953	11843.2088	-0.013	-1.1
y108	11971.2079	11971.3037	-0.096	-8.0
y111	12346.4641	12346.4654	-0.001	-0.1
y126	14132.236	14132.2612	-0.025	-1.8

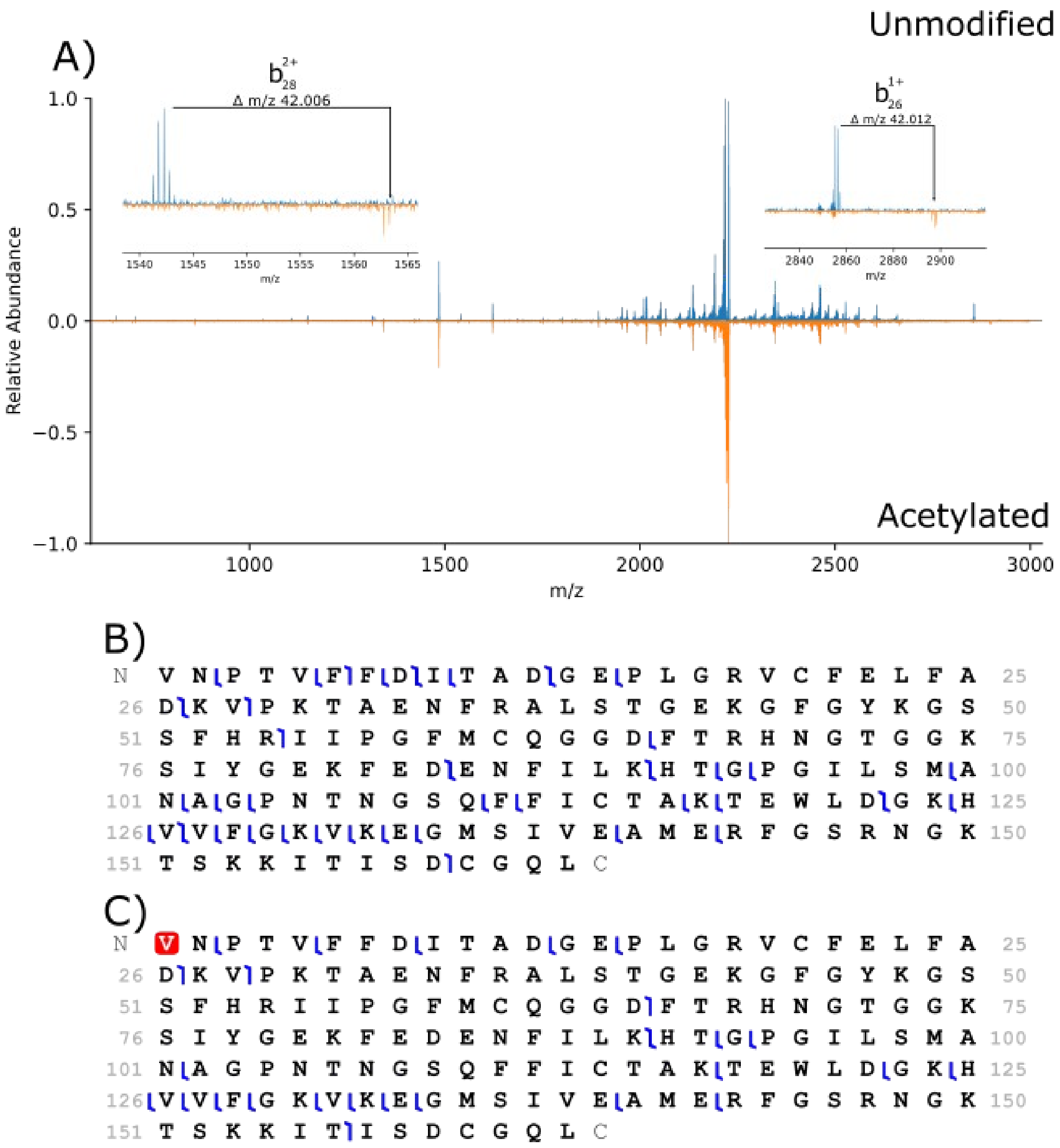

Figure S7: identification of proteoforms of PPIA. A) HCD (31% NCE (set to maximum charge of 5+)) of the 8+ charge state showing sequence fragments for both the unmodified (top, blue trace) and acetylated (bottom, orange trace) proteoforms of PPIA. Inset spectra show the 42 Da mass shift between key sequence fragments which reveal the presence of the modification. Each mass spectrum was acquired for 6 mins. B) Sequence coverage obtained for unmodified PPIA. C) Sequence coverage obtained for acetylated PPIA. The red square indicates acetylation

Table S8: Sequence ions observed for unmodified PPIA

Identity	Experimental Mass (Da)	Theoretical Mass (Da)	Error (Da)	Error (PPM)
b6	657.3504	657.3486	0.0018	2.7
b8	919.4396	919.4439	-0.0044	-4.7
b12	1319.6469	1319.6397	0.0072	5.4
b26	2853.3391	2853.3683	-0.0292	-10.2
b28	3080.5330	3080.5317	0.0014	0.4
b54	5933.9634	5933.9627	0.0007	0.1
b84	9176.4857	9176.4540	0.0318	3.5
b90	9920.8794	9920.8710	0.0084	0.8
b122	13280.3758	13280.4413	-0.0655	-4.9
b126	13701.5806	13701.6850	-0.1044	-7.6
b159	17312.4162	17312.5813	-0.1652	-9.5
y21	2295.2142	2295.2117	0.0025	1.1
y24	2626.3068	2626.3319	-0.0251	-9.6
y30	3242.6393	3242.6210	0.0183	5.7
y31	3371.6293	3371.6635	-0.0343	-10.2
y32	3499.7330	3499.7585	-0.0255	-7.3
y33	3598.7892	3598.8269	-0.0377	-10.5
y34	3726.9205	3726.9219	-0.0014	-0.4
y35	3783.9100	3783.9433	-0.0333	-8.8
y36	3930.9765	3931.0117	-0.0352	-9.0
y37	4030.0590	4030.0802	-0.0212	-5.3
y38	4129.1206	4129.1486	-0.0279	-6.8
y39	4266.2148	4266.2075	0.0073	1.7
y41	4451.3144	4451.3239	-0.0095	-2.1
y46	5095.5760	5095.6045	-0.0285	-5.6
y47	5223.6537	5223.6994	-0.0457	-8.7
y52	5758.8769	5758.9459	-0.0690	-12.0
y53	5905.9998	5906.0143	-0.0145	-2.4
y60	6604.2685	6604.3127	-0.0442	-6.7
y61	6661.2972	6661.3341	-0.0370	-5.5
y62	6732.3316	6732.3712	-0.0396	-5.9
y64	6917.3746	6917.4513	-0.0767	-11.1
y70	7515.7354	7515.7661	-0.0308	-4.1
y71	7572.7394	7572.7876	-0.0482	-6.4
y73	7810.8955	7810.8942	0.0013	0.2
y79	8555.2872	8555.3112	-0.0240	-2.8
y98	10679.1572	10679.3136	-0.1564	-14.6
y137	14878.2937	14878.3969	-0.1032	-6.9
y149	16225.9599	16226.0614	-0.1015	-6.3
y151	16412.1500	16412.1254	0.0246	1.5
y154	16699.1004	16699.2372	-0.1368	-8.2

y155	16812.2779	16812.3212	-0.0433	-2.6
y156	16927.1682	16927.3482	-0.1800	-10.6
y158	17221.4313	17221.4850	-0.0537	-3.1
y161	17518.5594	17518.6538	-0.0944	-5.4

Table S9: Sequence ions observed for N-acetylated PPIA.

Identity	Experimental Mass (Da)	Theoretical Mass (Da)	Error (Da)	Error (PPM)
b26	2895.3717	2895.3789	-0.0072	-2.5
b28	3122.5124	3122.5422	-0.0298	-9.5
b65	7094.398	7094.4621	-0.0641	-9.0
b90	9962.8416	9962.8816	-0.0399	-4.0
b150	16392.1128	16381.0475	-0.1590	-9.7
b156	17039.2852	17039.4489	-0.1637	-9.6
y21	2295.1935	2295.2117	-0.0182	-7.9
y24	2626.3327	2626.3319	0.0008	0.3
y30	3242.6397	3242.621	0.0187	5.8
y31	3371.6483	3371.6635	-0.0153	-4.5
y32	3499.7531	3499.7585	-0.0054	-1.5
y33	3598.8101	3598.8269	-0.0168	-4.7
y35	3783.9326	3783.9433	-0.0107	-2.8
y36	3931.0239	3931.0117	0.0121	3.1
y37	4030.0595	4030.0802	-0.0207	-5.1
y38	4129.1211	4129.1486	-0.0274	-6.6
y39	4266.1517	4266.2075	-0.0558	-13.1
y41	4451.3149	4451.3239	-0.0090	-2.0
y41	4451.334	4451.3239	0.0101	2.3
y46	5095.5716	5095.6045	-0.0329	-6.5
y62	6732.3753	6732.3712	0.0041	0.6
y70	7515.6858	7515.7661	-0.0803	-10.7
y70	7515.6948	7515.7661	-0.0713	-9.5
y71	7572.7404	7572.7876	-0.0472	-6.2
y73	7810.8036	7810.8942	-0.0905	-11.6
y73	7810.8952	7810.8942	0.0010	0.1
y149	16225.9619	16226.0614	-0.0994	-6.1
y151	16412.052	16412.1254	-0.0734	-4.5
y151	16412.0725	16412.1254	-0.0529	-3.2
y155	16812.294	16812.3212	-0.0273	-1.6
y158	17221.4336	17221.485	-0.0514	-3.0
y161	17518.5616	17518.6538	-0.0922	-5.3

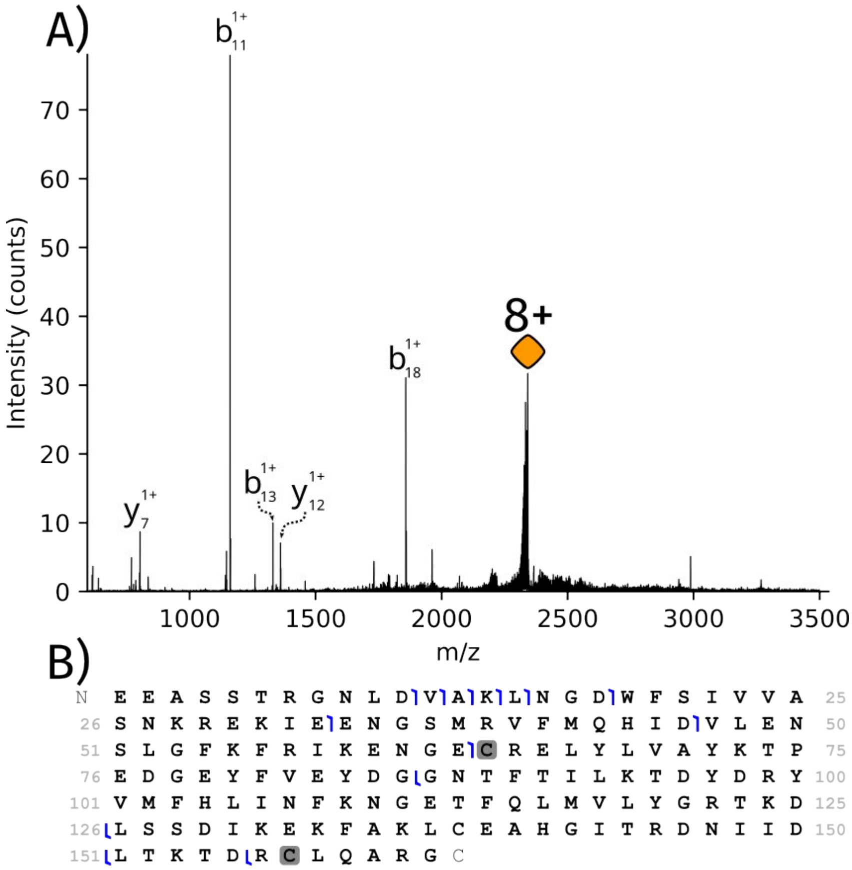

Figure S8: identification of MUP. A) HCD (NCE 40%) of the 8+ ions of MUP, data acquired for 15 mins. B) Sequence coverage obtained. The grey squares indicates a disulphide bond

Table S10: Sequence ions observed for MUP.

Identity	Experimental Mass (Da)	Theoretical Mass (Da)	Error (Da)	Error (PPM)
b15	786.4025	786.4048	0.002	-2.9
b11	1160.5141	1160.5178	0.004	-3.2
b12	1259.5818	1259.5862	0.004	-3.5
b13	1330.6187	1330.6233	0.005	-3.5
y12	1360.7146	1360.7240	0.009	-6.9
y7	802.4159	802.4226	0.007	-8.3
b14	1458.7131	1458.7183	0.005	-3.6
b46	1730.5181	1730.5226	0.005	-2.6
b63	1788.4014	1788.4030	0.002	-0.9
y76	1766.4952	1766.5083	0.013	-7.4
b33	1822.9338	1822.9370	0.003	-1.8
b18	1857.8874	1857.8937	0.006	-3.4
y37	2079.5750	2079.5873	0.012	-5.9

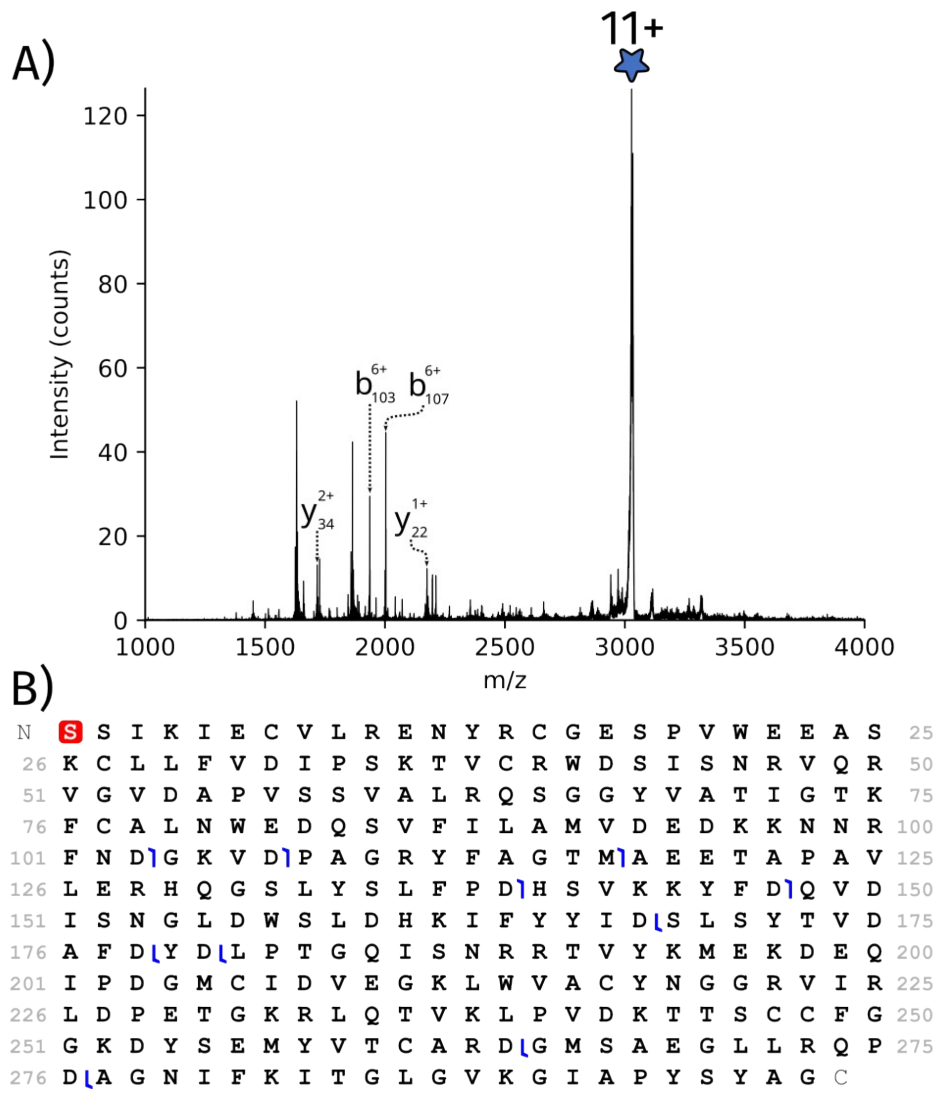

Figure S9: Identification of regucalcin (RGN). A) HCD of the 11+ charge state of RGN (NCE 42%), data acquired for 13 mins. B) Sequence coverage of the RGN1. The red square indicates N-acetylation.

Table S11: Sequence ions observed for RGN

Identity	Experimental Mass (Da)	Theoretical Mass (Da)	Error (Da)	Error (PPM)
b103	11607.6675	11607.7460	0.078	-6.8
b103	11607.6845	11607.7460	0.061	-5.3
b107	12006.8603	12006.9578	0.097	-8.1
b107	12006.9182	12006.9578	0.040	-3.3
b117	13058.6051	13058.4487	-0.156	12.0
b139	15469.6365	15469.6244	-0.012	0.8
b147	16474.0167	16474.1324	0.116	-7.0
y22	2196.1851	2196.1942	0.009	-4.1
y34	3450.7532	3450.7969	0.044	-12.7
y118	12906.2727	12906.4221	0.149	-11.6
y120	13184.4164	13184.5123	0.096	-7.3
y130	14282.9804	14282.9993	0.019	-1.3

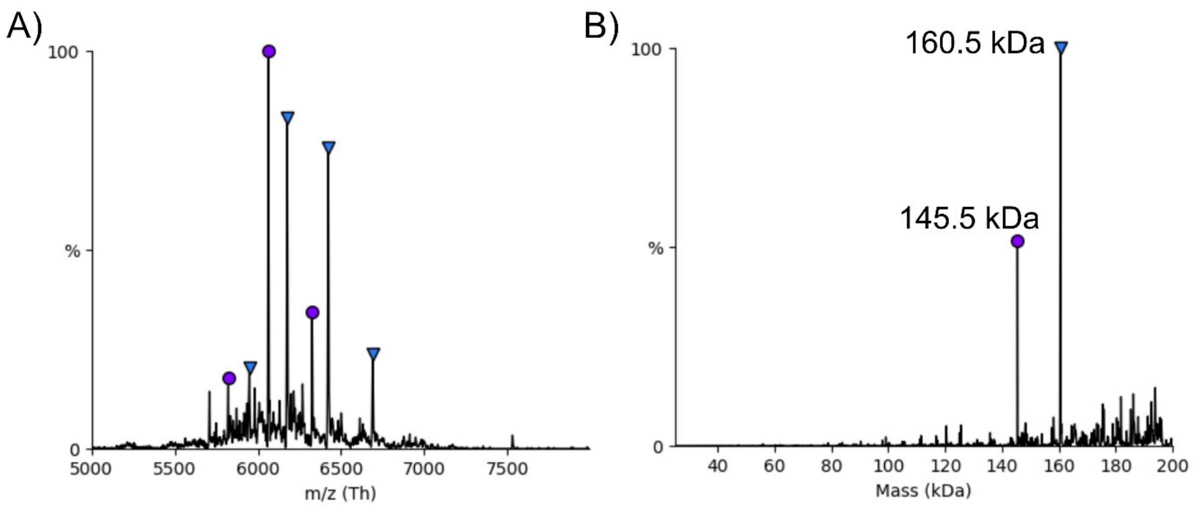

Figure S10: A) – Mass spectrum from LCMD extract of rat liver obtained with in-source collision energy =160 V; compensation scaling factor 16%, i.e., with mass spectrometer tuned for higher molecular weight species. B) Deconvoluted mass spectrum obtained following processing with UniDec software.

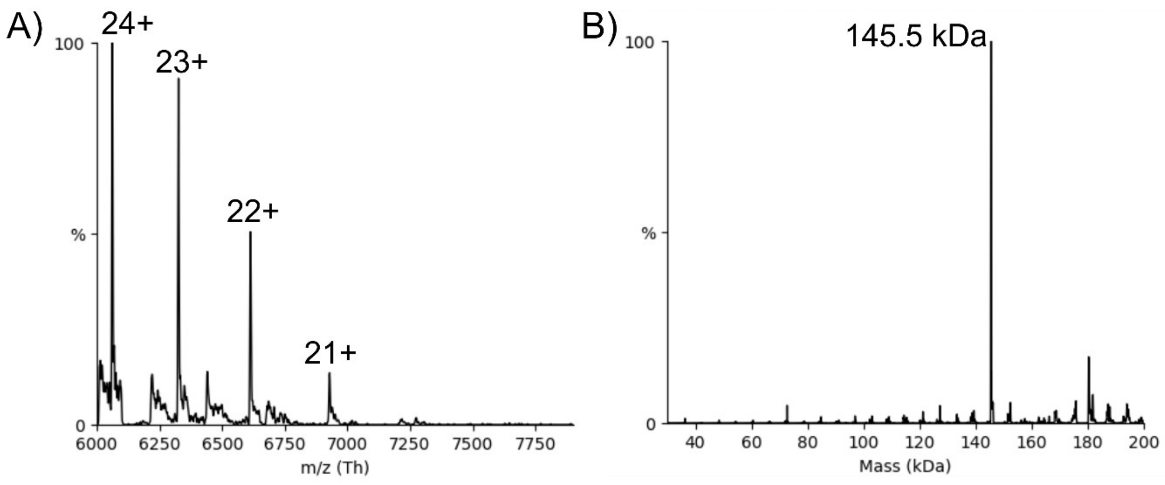

Figure S11: A) PTCR mass spectrum of the 24+ charge state of the LDHA monomer, B) Deconvolution of the PTCR spectrum using the UniDec software.

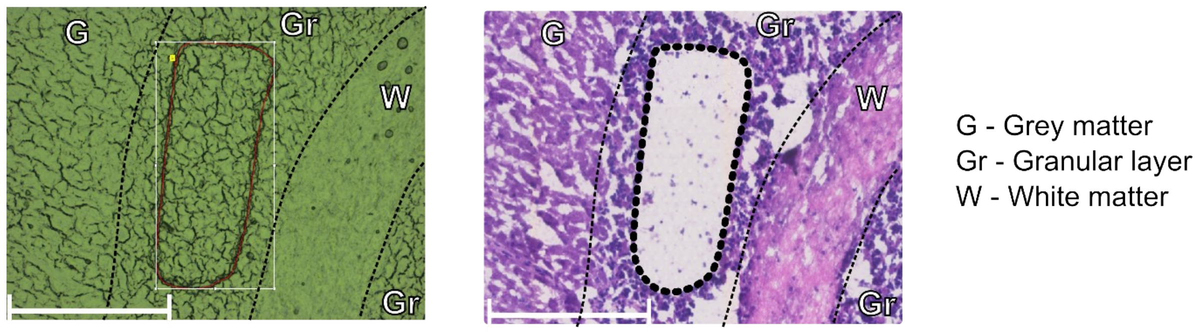

Figure S12: Optical images showing a region in the cerebellum of a rat brain A) before LCMD with the region in the granular layer defined to be captured and B) after LCMD where the tissue has been H&E stained. The three different regions (grey matter (G), white matter (W) and granular layer (Gr)) have been labelled. Scale bar = 200 µm.

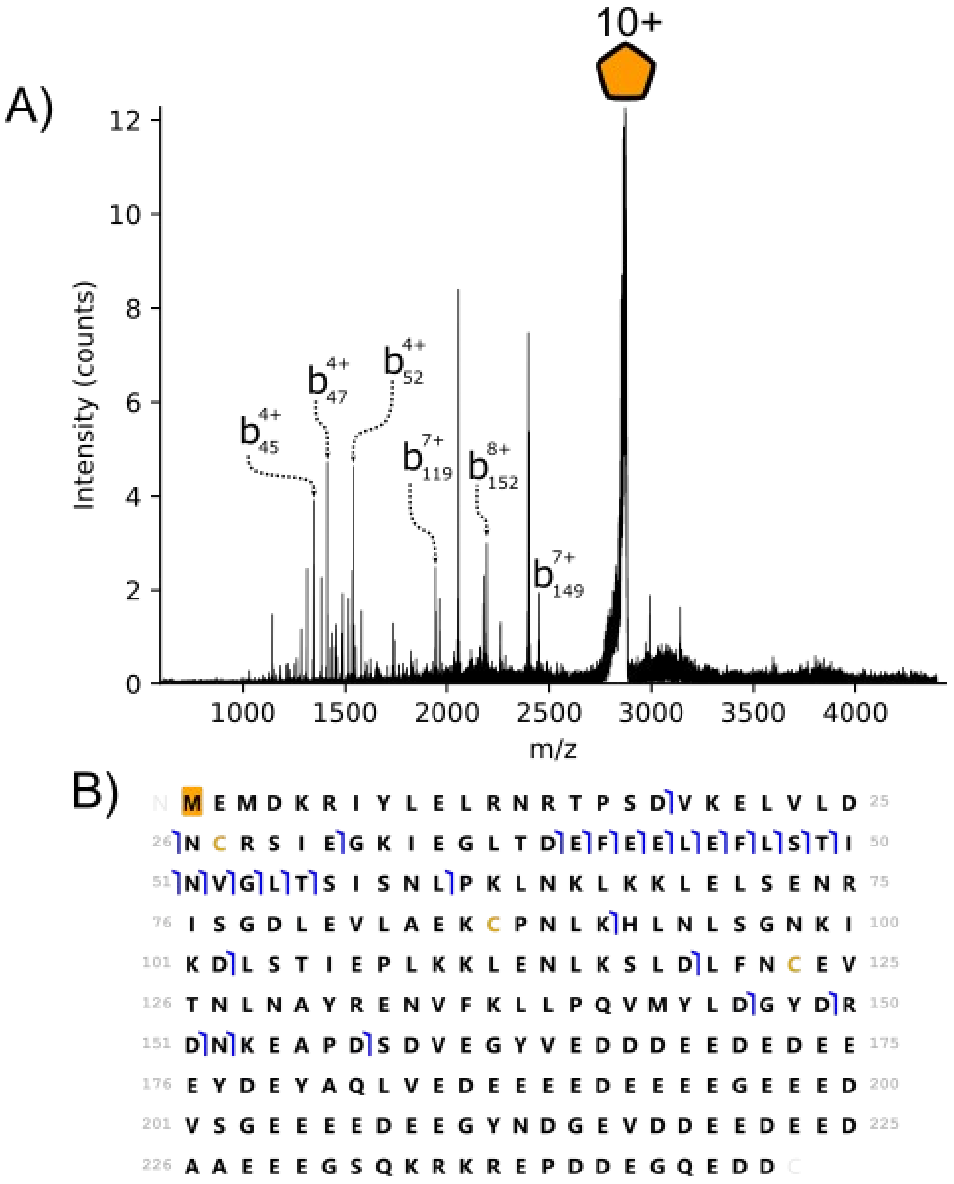

Figure S13: Identification of ANP32A. A) HCD fragmentation (NCE 40%) of the 10+ ions of ANP32A, data acquired for 20 mins B) Sequence coverage of ANP32A. The orange square indicates N-acetylation.

Table S12: Sequence ions observed for ANP32A.

Identity	Experimental Mass (Da)	Theoretical Mass (Da)	Error (Da)	Error (PPM)
b18	2290.123	2290.134	-0.011	-4.79902
b25	3086.588	3086.604	-0.0158	-5.11558
b31	3788.907	3788.923	-0.0162	-4.27224
b39	4602.337	4602.346	-0.0096	-2.08453
b40	4731.379	4731.396	-0.0168	-3.54775
b41	4878.459	4878.464	-0.0052	-1.06504
b42	5007.488	5007.507	-0.0188	-3.75137
b43	5136.526	5136.55	-0.024	-4.66876
b44	5249.611	5249.634	-0.0224	-4.26372
b45	5378.653	5378.676	-0.0232	-4.31012
b46	5525.714	5525.745	-0.0308	-5.56988
b47	5638.79	5638.829	-0.0392	-6.94687
b48	5725.831	5725.861	-0.03	-5.23573
b49	5826.883	5826.908	-0.0256	-4.3904
b50	5939.961	5939.993	-0.0316	-5.31629
b51	6054.02	6054.036	-0.0156	-2.57509
b52	6153.075	6153.104	-0.0292	-4.74249
b53	6210.103	6210.126	-0.0228	-3.66906
b54	6323.22	6323.21	0.0104	1.643695
b55	6424.232	6424.257	-0.0256	-3.98242
b60	6938.515	6938.532	-0.0176	-2.5351
b91	10439.49	10439.52	-0.0294	-2.8146
b102	11659.14	11659.19	-0.0534	-4.57772
b119	13581.28	13581.31	-0.028	-2.0606
b146	16808.86	16808.92	-0.0595	-3.53831
b149	17143.98	17144.03	-0.0539	-3.14267
b151	17415.1	17415.17	-0.0632	-3.62735
b152	17529.12	17529.21	-0.0896	-5.10914
b157	18069.42	18069.46	-0.0408	-2.25695

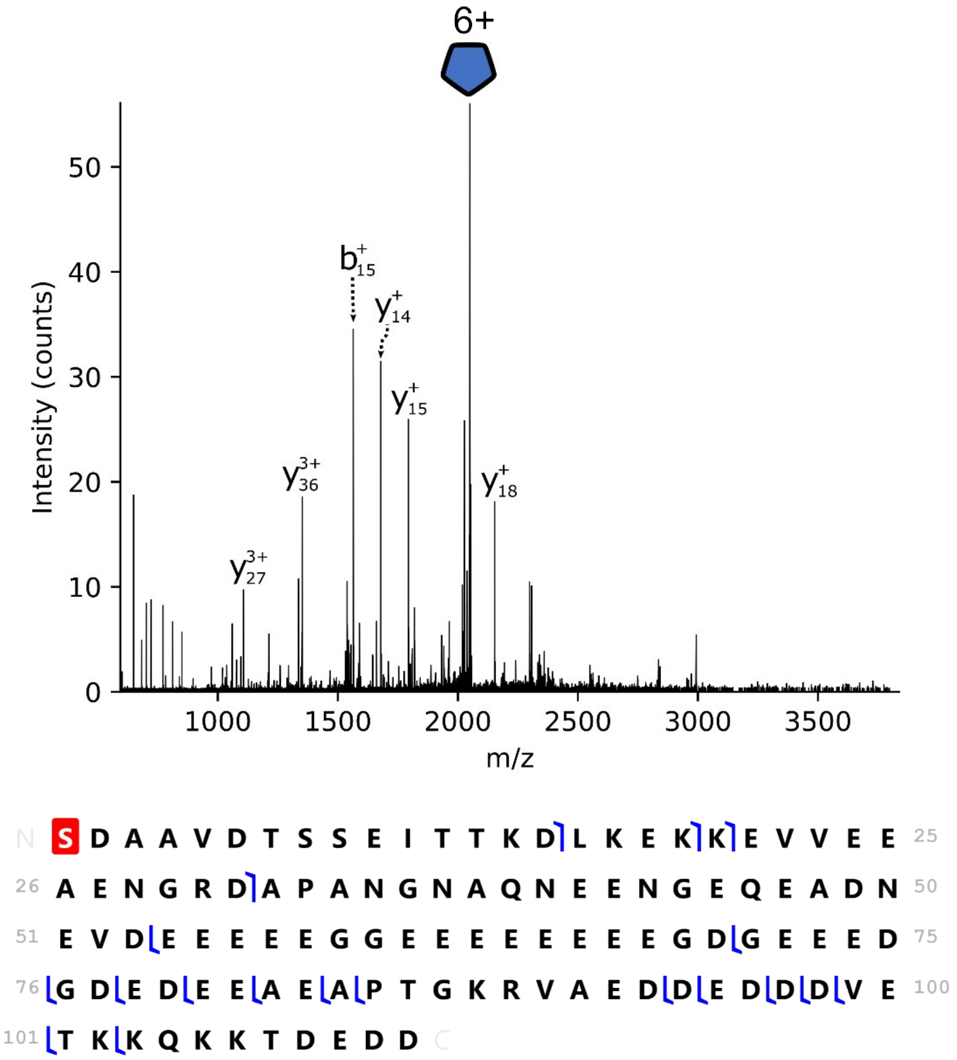

Figure S14: identification of PTMA. A) HCD fragmentation (NCE 35%) of the 6+ ions of PTMA, data acquired for 5 mins B) Sequence coverage obtained for PTMA. The red square indicates N-acetylation.

Table S13: Sequence ions observed for PTMA

Identity	Experimental Mass (Da)	Theoretical Mass (Da)	Error (Da)	Error (PPM)
y9	1105.529	1105.532	-0.0035	-3.16303
y11	1334.671	1334.675	-0.0045	-3.36908
b15	1562.698	1562.702	-0.0041	-2.62198
y13	1562.781	1562.786	-0.005	-3.19737
y14	1677.806	1677.813	-0.0066	-3.93135
y15	1792.835	1792.84	-0.0052	-2.89881
y17	2036.904	2036.91	-0.0054	-2.64977
y17	2036.911	2036.917	-0.0062	-3.04083
b19	2061.019	2061.026	-0.007	-3.39307
y18	2151.929	2151.936	-0.0075	-3.48361
b20	2189.115	2189.121	-0.0054	-2.46449
y27	3105.426	3105.437	-0.011	-3.5399
y27	3105.434	3105.444	-0.0099	-3.18487
y28	3176.471	3176.474	-0.0032	-1.00677
y30	3376.545	3376.554	-0.0084	-2.48627
b31	3416.647	3416.658	-0.0104	-3.04213
y32	3634.633	3634.639	-0.006	-1.64988
y32	3634.633	3634.646	-0.0129	-3.54625
y34	3878.699	3878.708	-0.009	-2.31916
y36	4050.744	4050.757	-0.013	-3.20769
y36	4050.751	4050.764	-0.0132	-3.25623
y41	4609.904	4609.933	-0.0288	-6.24467
y41	4609.93	4609.94	-0.0102	-2.21117
y58	6573.555	6573.585	-0.0306	-4.65287
